# Face Identity Tests and Inclusion Criteria in Self-Identified Acquired Prosopagnosia

**DOI:** 10.3390/brainsci16090923

**Published:** 2026-08-29

**Authors:** Edwin J. Burns, John R. Towler

**Affiliations:** School of Psychology, Swansea University; Swansea SA2 8PQ, UK; j.r.towler@swansea.ac.uk

**Keywords:** diagnosis, criteria, prosopagnosia, single-case analysis, DSM-5

## Abstract

**Highlights:**

**What are the main findings?**

- Roughly half of people with self-identified acquired prosopagnosia failed to meet widely recommended cognitive test-based diagnostic criteria for prosopagnosia.
- The PI-20 prosopagnosia questionnaire combined with a famous faces test was the most sensitive package for detecting impairment in people who self-identify as having acquired prosopagnosia.

**What are the implications of the main findings?**

- Requiring impairment on two cognitive tests excludes acquired prosopagnosia cases that exhibit severe problems with familiar face recognition.
- Acquired prosopagnosia research must become more inclusive, and guided further by patients’ self-reports, in order to improve our understanding of their daily difficulties and our tests’ validity.

**Abstract:**

Background/Objectives: Prosopagnosia is characterised by severe difficulties recognising facial identity. While many self-identified developmental cases fail to meet widely used cognitive task-based criteria for inclusion in research, the extent to which this is an issue in acquired prosopagnosia is unknown. We therefore assessed the sensitivity of widely used inclusion cutoffs and face-processing tests in 14 self-identified British acquired prosopagnosia cases. Results: We found the −2 SD cutoff did not detect impairment in 9/11 (82%) individuals on the Cambridge Face Perception Test, 5/11 (45%) of cases on the Cambridge Face Memory Test, and 2/14 (14%) on the Famous Faces Test. Requiring impairments on two memory tasks at −2 SD or −1 SD resulted in at most 8/14 (57%) and 5/14 (36%) of cases not meeting the criteria, respectively. A more liberal approach, which additionally allowed for inclusion using a single test score at −2 SD, performed better by excluding at most 2/14 (14%). Given that the Famous Faces Test alone at the −2 SD level performed identically to our liberal approach, we suggest it could be used if an objective assessment is deemed necessary. Conclusions: We extend the recent suggestions in the literature that self-identified prosopagnosia cases who fail to meet cognitive test criteria warrant further investigation to explain the reasons for their self-reported status. We therefore advocate a more inclusive approach to prosopagnosia research.

## 1. Introduction

Prosopagnosia is defined as a cognitive deficit characterised by severe difficulties recognising faces [1,2]. People with this condition will often fail to identify people personally known to them, such as co-workers, neighbours, friends, and even close family members. Prosopagnosia can come in two forms. This first is developmental prosopagnosia (DP), which is lifelong [1,3], affecting 2–5% of the general population [4,5]. It can run in families [6,7,8], suggesting a possible hereditary component, which could explain the wide range of face-related neural atypicalities these cases exhibit [9,10,11,12,13,14,15,16,17,18,19,20,21,22]. The second form is acquired (AP), often occurring after a head injury, stroke, or infection has typically damaged areas of the brain important for face recognition [2,23,24,25].

### 1.1. The Traditional Approach to Including Prosopagnosia for Research

Both the developmental and acquired forms have traditionally been identified for inclusion in research studies using cognitive assessments of face processing, having to score at least −2 SDs below neurotypical means on two such tasks [26,27,28,29,30]. One test often deemed essential for inclusion is the Cambridge Face Memory Test, where participants learn six unfamiliar faces over a series of viewpoints before picking each target out of a line-up containing two novel lures [31]. However, recent papers have shown that roughly 35–56% of self-identified DP cases will fail to meet the CFMT’s −2 SD inclusion cutoff [27,32,33]. Moreover, these excluded cases that exhibit group-level impairments on multiple face-processing tasks [27], with meta-analyses further confirming their presence [34]. We have argued these objective difficulties with face recognition validate such cases’ self-identified prosopagnosia status [27], even though they fail to meet the most prevalent criterion employed by researchers.

As mentioned, impairment at the −2 SD level on the CFMT alone is rarely sufficient to allow a self-identified prosopagnosic to be included in research. Individuals often need to score more than −2 SDs below a neurotypical mean on a Famous Faces Test (FFT), too [27,30]. Although not standardised, such tasks will normally show a series of single still images of highly famous celebrities and ask participants to identify each face by name or some form of identifiable semantic information [27]. However, like the CFMT, between 25 and 74% of DP cases will fail to meet the −2 SD cutoff on this task despite reporting highly abnormal levels of symptoms [27]. Moreover, research exclusions inevitably increase when cases are required to score below the −2 SD cutoff on the combination of the CFMT and FFT, as most researchers require [27], including, as is often the case, in AP [35,36]. We call this the conservative form of the traditional cognitive task-based approach to classification of prosopagnosia for research purposes (Table 1).

Less frequently, a face-matching task called the Cambridge Face Perception Test (CFPT) can be used to help classify individuals as having prosopagnosia [37]; however, it is rarely a requirement [27,30]. This is because it is unclear whether prosopagnosia can always be defined as a perceptual disorder (i.e., all faces look alike to those with the condition) rather than an issue with memory [38]. The CFPT involves rearranging a series of visually morphed face images in order of similarity to a target face [37]. When this test is used to identify prosopagnosia for research purposes, cases will often have to score more poorly than −2 SDs below the neurotypical mean on at least two out of three (i.e., CFMT, FFT and CFPT) assessments [27,30]. This means that even if a patient fails to meet the −2 SD criteria on a single test out of the CFMT or FFT, they may still have an opportunity to be judged atypical using the CFPT, thus still meeting the −2 SD impairment on two tests of identity processing that many researchers recommend. However, reanalysing our previous data [34] reveals even this approach still excludes around 60% of self-identified DP cases from being included in research.

### 1.2. The Adapted and Liberal DSM-5 Approaches to Identifying Prosopagnosia

The high frequency of self-identified cases failing to meet the −2 SD inclusion criteria on two cognitive tests is now well established in DP across multiple papers [26,27,32,33]. Moreover, these cases exhibit objective impairments at the group level across multiple cognitive tests of face processing [27]. Some authors have therefore argued that these inclusion criteria are too conservative [27,30]. As a solution, some have advocated using an adapted DSM-5 approach to neurocognitive disorders (i.e., acquired cognitive impairment, otherwise known as neurocognitive disorders; [39]) when identifying prosopagnosia [30,39]. This liberalises the −2 SD on two cognitive tests requirement to −1 SD, thus reducing the frequency of cases that would be excluded from research. Moreover, some authors have proposed that these must be two tests of face recognition [30], i.e., the CFPT would be unsuitable. We therefore call this approach the Adapted DSM-5 method.

However, the DSM-5 has been criticised for imperfectly discriminating between self-identified DP and neurotypicals’ face recognition abilities because of substantial overlaps in the two groups’ performance distributions [27,34]. For example, the DSM-5 will classify some neurotypicals as abnormal [40,41] but also excludes 30–38% of DP cases who are objectively impaired at the group level on multiple cognitive assessments [27,34], with meta-analyses corroborating these objective difficulties [34]. Moreover, the Adapted DSM-5 requirement of impairment on two tests failed to detect impairment in many patients who are highly abnormal on a single test (e.g., up to −7 SDs below a neurotypical mean on the FFT; [34]). This is because tasks like the CFMT may lack the sensitivity for detecting the cognitive processes that are primarily impaired in developmental prosopagnosia (e.g., familiar face recognition) and lack the reliability to classify prosopagnosia consistently (i.e., cases’ status as prosopagnosic or not changes from one day to the next) [4,34]. Given that many DP cases exhibit severe problems on a single test, Burns (2024) [34] suggested that if objective tests must be used for identifying prosopagnosia in research, then a liberalised version of the DSM-5 approach that adds the option of including at the −2 SD level on a single test would overcome some of these problems.

### 1.3. Do Current Cognitive Test Criteria Exclude AP Cases from Research?

Remarkably, while self-identified cases who fail to meet objective, single-case criteria are now well established in DP, such occurrences have not been systematically identified in people who self-identify as having acquired prosopagnosia. Importantly, there are hints in the literature that such individuals exist. We anecdotally reported on a self-identified acquired case who, following a stroke, reported severe problems with faces in daily life at interview but then failed to meet cognitive-task-based inclusion criteria [27]. This is corroborated by examples of acquired cases scoring one or two trials correctly above the −2 SD cutoff on the CFMT [42,43]. Fysh and Ramon [44] also found an acquired prosopagnosia patient who exhibited neurotypical accuracy rates on four face-processing tests. Finally, after reviewing historical medical records, Josephs and Josephs [45] identified examples of acquired prosopagnosia cases performing normally on objective tests. Unfortunately, no details were provided on the tests used or exactly how many patients this affected [45]. While these findings suggest individuals self-identifying as having AP can perform normally on cognitive tests of face recognition, they are rarely discussed in the literature. This means it is unknown if the rates of such exclusions can reach as high as those found in DP, i.e., up to 85% of self-identified cases failing to meet inclusion criteria [34].

Such a finding would be important for several reasons. First, it would suggest there may be an important gap between the types of problems people with prosopagnosia report experiencing in the real world versus those that we can detect through cognitive tests. For example, all individuals who self-identify as DP report extremely severe levels of symptoms but often only exhibit mild difficulties, if any, on the CFMT [34]. If prosopagnosics’ abilities to self-identify are accurate but researchers exclude these individuals from research due to their cognitive test results, then it is inevitable we will never learn more about these individuals’ problems. Moreover, we will be unable to be confident about the validity of commonly used tests to identify acquired prosopagnosia. For example, while the CFMT’s validity and reliability have been tested in many DP papers (e.g., [26,27,34]), similar work is lacking in self-identified acquired prosopagnosia.

Moreover, if we only study AP patients who perform the most poorly on face-processing tests, we will inevitably overestimate effect sizes of impairments on these measures and other areas of cognition that may be correlated with them (e.g., emotion processing, reading, object recognition [27,34]). Pre-morbid face recognition ability (i.e., prior to the accident or injury that caused the prosopagnosia) is almost never known for individuals who have acquired prosopagnosia in adulthood. This raises additional issues, for example, whether an individual may have had face recognition abilities that would have been measured at 1 SD above the mean prior to a stroke and now scores −1 SD below the mean on face-processing tasks. This injury represents a substantial (−2 SD) qualitative loss of face-processing ability that would clearly be noticed by the individual, and yet they may still perform better than many control participants due to the substantial variability within the general population [27,34,46]. In this instance, the AP patient may only be performing within the normal range due to remedial strategies but still be functioning abnormally (i.e., failing to recognise personally familiar faces) in daily life. Given these reasons why many AP cases may fail to meet research related inclusion criteria, it is crucial to transparently document self-reported acquired prosopagnosics’ test performance to find out what is normative in this understudied group.

This highlights a broader problem of establishing ground truth in prosopagnosia. Self-reporting may not, by itself, necessarily establish the presence of prosopagnosia, but neither should performance on any single cognitive test or conventional cutoff be assumed to provide ground truth. Cognitive tests provide standardised measurements of performance under laboratory conditions, whereas self-reporting provides information about perceived difficulties encountered in everyday life. Where these sources of information disagree, the discrepancy itself may therefore be informative [27,34]. Our approach is not to assume that self-identification is necessarily correct, but to take reports of a marked acquired deterioration in face recognition seriously enough to characterise where these individuals fall on commonly used cognitive measures, rather than excluding them *a priori* on the basis of those same measures.

We therefore wanted to assess in an exploratory study how many self-identified AP cases fail to meet the common research inclusion cutoff of −2 SDs on three widely used neuropsychological assessments for face processing: the CFMT, CFPT and the FFT. Prior work has suggested that self-identified DP failed to detect impairment in around 20%, 50% and 80% on the FFT, CFMT and CFPT, respectively [27], so we were curious if these were replicated in a self-identified acquired sample. Moreover, we wanted to compare the extent to which the different inclusion approaches outlined in Table 1 are effective at detecting atypicality in self-identified acquired prosopagnosia. These included a variety of cognitive task-based inclusion methods: the two-tests approach (−2 SDs on the FFT and CFMT), the two-out-of-three tests approach (−2 SDs on two of CFMT, FFT or CFPT), an Adapted DSM-5 approach (−1 SD on CFMT and FFT), and an exploratory Liberal DSM-5 approach (i.e., impaired at −1 SD on two tests or impaired at −2 SD on a single test).

## 2. Materials and Methods

### 2.1. Participants

The self-identified AP sample comprised 14 cases whose ages ranged between 39 and 76 years old (M = 57.6, SD = 11.1), with six females and eight males. All the cases reported acquiring prosopagnosia following a brain injury or stroke and reported severe difficulties with faces in daily life. All the participants were White British, none had a diagnosis of autism or dementia, and all reported normal or corrected to normal visual acuity. All the cases reported severe difficulties in daily life and completed a Famous Faces Test [32]. Only eleven of the 14 AP cases additionally completed the Cambridge Face Memory Test and Cambridge Face Perception Tests. While three of our 14 AP cases did not complete these tasks, we felt, given how rare these cases are, it was important to include their data where possible. The participants were drawn from a broader programme of prosopagnosia research at Swansea University. Following public engagement and media coverage of this work (Evans, 2019, https://www.bbc.co.uk/news/uk-wales-47304678, accessed on 27 August 2026), several hundred individuals contacted the research group because they experienced difficulties recognising faces [47,48]. Most described lifelong difficulties consistent with developmental prosopagnosia, whereas a much smaller group reported a marked deterioration in face recognition following brain injury or stroke. The 14 individuals reported here were drawn from this latter group and contacted the research team because of these acquired everyday difficulties.

As part of this wider research programme, all of the present participants completed the 20-item Prosopagnosia Index (PI20), a standardised self-report measure of difficulties with face recognition. All the AP participants’ PI20 scores indicated severe difficulties recognizing faces in daily life, with Z-scores ranging from −2.03 to −4.7 (*M* = −3.19, *SD* = 0.77). These scores are reported in Table 2. Some participants also informally described neurological assessment and brain imaging in informal correspondence with the research team; however, detailed clinical records were not systematically collected, and these reports are therefore not treated here as an independently verified clinical diagnosis.

### 2.2. Procedure and Materials

The participants completed the PI20 self-report questionnaire [49], along with a battery of cognitive tests that have been used to classify prosopagnosia for research purposes. These included the Cambridge Face Memory Test (CFMT; [31]), the Cambridge Face Perception Test (CFPT; [37]), and a Famous Faces Test (FFT) that has been reported on before [32].

The Cambridge Face Memory Test (CFMT; [31]) has been the most commonly used test for classifying developmental prosopagnosia [27]. Participants study six target identities before selecting them in a lineup containing two lures. The targets are presented in noise to increase the difficulty in a final section. The CFMT comprises 72 trials, with one point given for each correct trial. This test is commonly used to assess face memory in neurotypical [50,51,52,53], neurodivergent [54,55] and DP cases [56,57,58]. We computed a −2 and −1 SD CFMT threshold using norms from the initial paper [31].

In the Cambridge Face Perception Test [37] participants rearrange six faces in order of similarity to a target face. These faces were morphed in varying degrees between the target and a second facial identity. Scores are recorded as errors from where the target face should have been placed in order of its similarity to the target. The version we used here contained eight upright trials, and none of the eight inverted trials were found in the original version. Normative scores were taken from the CFPT’s original paper [37].

The participants completed a famous faces test (FFT) that has been reported on in previous work [32]. There were two versions: one for participants under 35 years old, and another for those above that age, comprising famous faces known to the respective age groups. Both presented 60 trials of a celebrity’s face image. The participants then attempted to identify each celebrity using their name or related semantic information to demonstrate that they recognised the celebrity. The participants were then shown information of the individual pictured and had to indicate whether they knew them. We used this value to correct the participants’ scores as a percentage of correct responses for the famous individuals that they knew.

The 20-item Prosopagnosia Index (PI20; [49]) quantifies self-reported difficulties with face recognition in everyday life. Higher scores indicate greater self-reported face-recognition difficulty.

Ethical approval was granted by Swansea University Ethics Review Board, with all work carried out in accordance with the 1964 Helsinki Declaration on human testing. All the participants gave their informed consent, including for anonymised data derived from their participation to be published. The data can be downloaded from the OSF (https://osf.io/4q7az/files/kj5xf, accessed on 27 August 2026).

## 3. Acquired Prosopagnosia Cases’ Methods and Results

### 3.1. Single Cognitive Test Results

We were first interested in how the AP cases performed on the CFMT, given that it is the most widely used standardised test to classify prosopagnosia [27,34]. CFMT accuracy in our AP sample ranged from −0.24 to −4.29 SDs below the neurotypical norm. Although we should use caution interpreting these rates given the small sample size, it is worth noting that the percentage of cases that did not exhibit impairment on the CFPT is similar to prior work on developmental prosopagnosia [26,27,32,33], with 46% of acquired cases (5/11) failing to meet the single case −2 SD requirement. When all the AP cases were combined, their average level of CFMT impairment was −2.05 SDs. Again, while we urge caution comparing these results to the developmental literature, this is remarkably similar to the mean CFMT impairment in self-identified DP cases (−1.79 SDs; [27]). Please note, as Duchaine and Nakayama [31] did not report CFMT subsection norms, we used Hedley et al.’s (2011) data to analyse the self-identified APs’ subsection performance [59]. This revealed 6/11 APs as abnormal at −2 SD in the learn phase and only two as atypical in the test and noise phases, respectively, with one of these AP cases atypical in both these latter phases. This suggests the CFMT’s learning phase is the most sensitive for detecting difficulties with faces at the −2 SD level in self-identified AP cases.

Table 2 also shows that the self-identified AP cases’ FFT scores ranged from −0.03 to −9.41 SDs. Only 14% (2/14) did not meet the −2 SD cutoff on this task. Although we should use caution comparing these rates to the DP literature given our comparatively small sample size, this is close to the highest end estimates of individually atypical FFT scores when testing self-identified DP cases (25%; [32]). The mean level of FFT accuracy in self-reported AP cases was −4.71 SDs below neurotypicals.

As expected, the CFPT detected much weaker levels of impairment than the two memory tasks, with scores ranging from 1.02 down to −2.14 SDs. Overall, only 18% (2/11) of self-identified AP cases met the −2 SD cutoff for atypicality. Again, while we should be cautious in extrapolating too much from the comparison, it is close to self-identified DP cases (i.e., 20–23%; [27,32,34]). Despite this, the average level of CFPT impairment in the AP group was −0.77 SDs, which appears milder than the −1.28 SDs reported in self-identified DP cases [27,34].

### 3.2. Approaches to Including Self-Identified AP in Research

#### 3.2.1. Two-Tests Approach

Table 2 also illustrates how many AP cases would have been classified as having AP, and therefore included in research papers, using different inclusion criteria. The two-tests approach (first column Table 2) is one of the mostly widely used in prosopagnosia research, requiring an individual to score more than −2 SDs below a neurotypical mean on the FFT and CFMT [30]. Out of the 11 cases who completed all the tests, this approach would have only classified 55% (6/11) acquired prosopagnosia cases. Of these, a lack of impairment in all the cases was due to the CFMT, even though their FFT scores would have been considered statistically abnormal using a single-case approach (i.e., poorer than −2 SDs). However, the FFT was not perfect: at least two out of the three self-identified AP cases who only completed the FFT also failed to meet inclusion criteria using this approach. This means the maximum number included in research using this method would have been 7/14 (50%) when the cases with incomplete data were included.

#### 3.2.2. Two of CFPT, CFMT and FFT

The second column in Table 2 illustrates the next inclusion criteria for research into prosopagnosia, where atypicality at the −2 SD level on two out of three tests (i.e., CFMT, CFPT, and FFT) would suffice. This approach is often used in DP research, e.g., [22,27,56,60,61,62,63,64]. Here, it resulted in no improvement in inclusion rates for the 11 cases who completed all tasks, with only 55% (6/11) meeting criteria. This was because only two cases were atypical on the CFPT, and even then, their CFMT impairments were the most severe in the whole AP group, and thus, they were already afforded an AP classification using the previous approach. It should be noted that given the strong positive correlation between the AP cases’ CFPT and FFT z-scores [*r* = 0.68, *p* = 0.021], it is likely that two of the cases with incomplete data would also have failed to meet inclusion criteria. This is due to the fact they performed the best of all the cases on the FFT, scoring close to the neurotypical mean.

#### 3.2.3. Adapted DSM-5 Approach

Given the high rates in which self-identified DP cases fail to meet objective criteria when using the approaches above, some authors have recommended including prosopagnosia in our research using a −1 SD cutoff on two tests of face memory [30]. This is adapted from the DSM-5 approach to diagnosing neurocognitive disorders (i.e., acquired cognitive impairment), and we call this the Adapted DSM-5 approach, with its classification rates presented in the third column of Table 2. Using this, we found that the inclusion rates improved in our cases who completed all the tests to 9/12 (82%). However, adding in the three cases with incomplete data reduced this to at most 10/14 (71%). Thus, 29% of self-identified AP cases would not be included in research using the Adapted DSM-5 approach. While we should note caution given the small sample size, this is remarkably similar to the 30–38% of self-identified cases that exhibited no objective impairment when using the same method in developmental prosopagnosia [34].

#### 3.2.4. Liberal DSM-5 Approach

**Figure 1:**
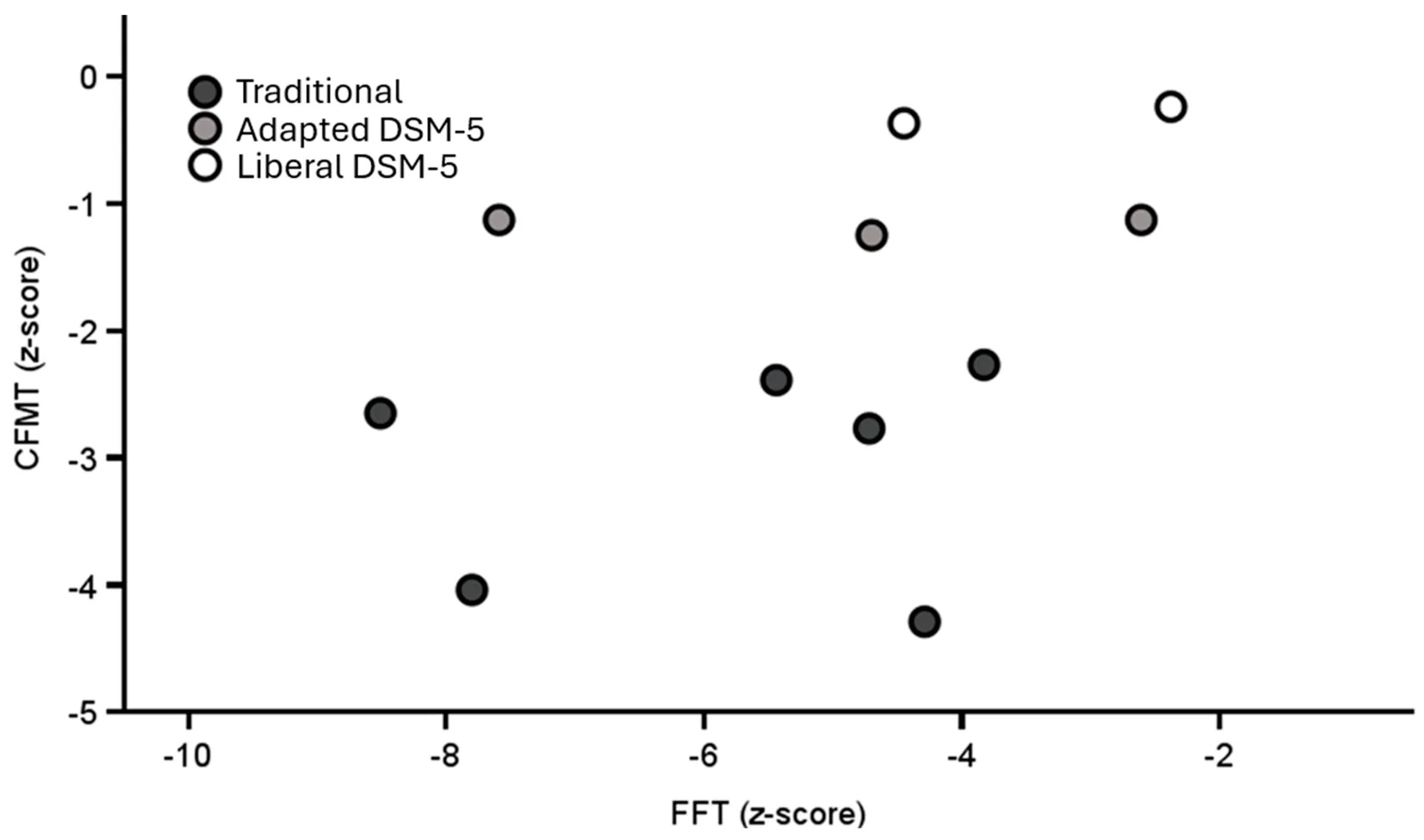
Illustration of the main approaches to including acquired prosopagnosia. Six of the 11 cases were classified as AP using the two-test approach (i.e., impaired at the −2 SD level on two cognitive tests). This increased to 9/11 using the Adapted DSM-5 approach (i.e., impaired −1 SD on CFMT and FFT), with all cases captured using the Liberal DSM-5 method (i.e., impaired at −1 SD on two tests or −2 on a single test). Please note, there were three cases with only FFT data who could not be plotted (see Table 2).

**Figure 2:**
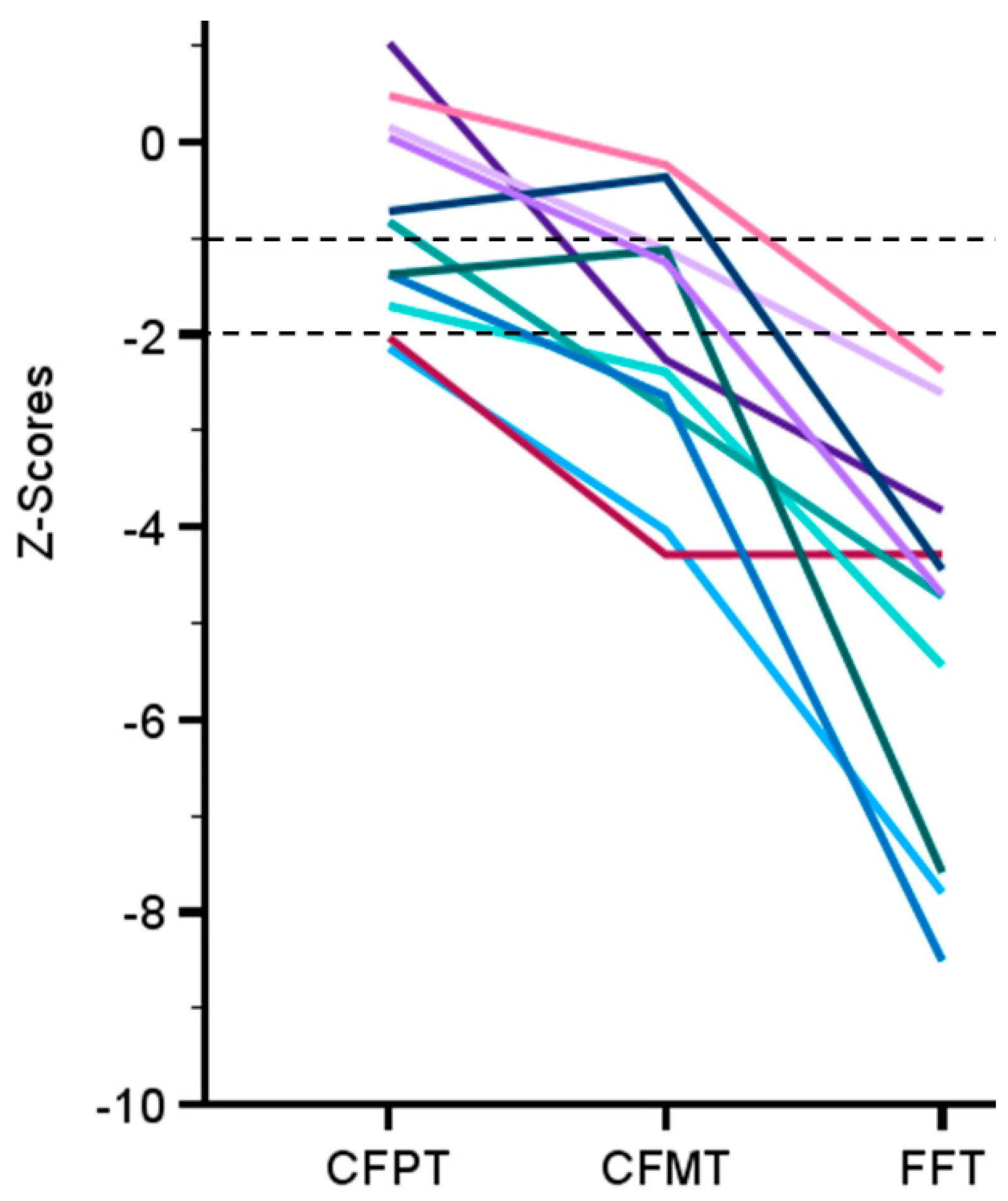
Illustration of z-scores from AP cases who completed all three tests. Dashed lines indicate the −1 and −2 SD cutoffs. Distinct colours represent individual AP cases.

We recently showed that the Adapted DSM-5 approach did not detect impairment in many developmental prosopagnosia cases who were highly abnormal (i.e., poorer than −2 SDs) in their single test scores [34]. As a result, we recommended that if the DSM-5 method must be used, then one method for making it more inclusive is to add an option to include prosopagnosics at the −2 SD level on a single cognitive test to ensure these cases are not excluded from our research [34]. We call this the Liberal DSM-5 approach, and it improved the Adapted DSM-5 inclusion rates for patients who completed all the tests from 9/11 (82%) to 11/11 (100%), with these rates graphically illustrated in Figure 1. We also show how these scores connect across all measures in Figure 2. Including the cases who had incomplete data showed the Adapted DSM-5 approach only classified at most 10/14 (71%), while the Liberal approach improved this to 12/14 (86%). Importantly, the improved classification rates by the Liberal DSM-5 approach remained the same if we had simply used the FFT at the −2 SD level alone. This suggests that other tasks, like the CFMT and CFPT, may cause exclusion of self-identified acquired prosopagnosia for research purposes.

## 4. Discussion

Prior work has shown that many self-identified DP cases do not meet objective, single-case criteria to be included in research when assessed through widely used face-processing tasks [27,32,33]. While a few such cases in AP have been reported in the literature [44,45], this issue had not been assessed in a group of people who self-identify as having AP. In our exploratory study, we showed that cases excluded from research arose in roughly 50% of self-identified acquired cases when we used the traditional −2 SD cognitive task-based approaches. Liberalising the cutoffs to −1 SD on two face memory tests (i.e., the Adapted DSM-5 method) still excluded 18–29% of self-identified AP cases, some of whom were highly abnormal in their FFT scores. Adding an additional classification option of impairment at the −2 SD level on a single test (i.e., the Liberal DSM-5 approach) improved the identification of abnormality to 0–14%.

It is important to note that the present participants were not assessed using a structured clinical interview, and our neurological characterisation of their acquired difficulties was limited. However, the available PI20 scores provide a standardised measure of self-reported everyday face-recognition difficulty, alongside the participants’ reports of marked deterioration in face recognition following brain injury or stroke. These sources of information do not, by themselves, establish an independently verified diagnosis of acquired prosopagnosia, but neither does performance within a conventional range on a cognitive test establish the absence of a meaningful acquired decline.

One reason for such a discrepancy is that the premorbid face-recognition ability is almost never known. An individual who performed substantially above the population mean before injury could experience a large and noticeable deterioration while still scoring within, or only slightly below, the conventional range on a cognitive test. Acquired failures of familiar-face recognition may also be particularly salient in everyday life, where failures to recognise co-workers, friends or family members can become apparent through repeated social interactions. The present data cannot determine whether discrepancies between self-report and cognitive-test performance reflect premorbid ability, compensatory strategies, limitations in test sensitivity, metacognitive factors, broader neurological impairment, or some combination of these factors. Rather than assuming that either self-report or cognitive-test performance provides definitive ground truth, we suggest that this discrepancy is itself an important subject for further investigation.

### 4.1. Considerations When Conducting Acquired Prosopagnosia Research

Self-identified AP participants, where abnormality cannot be detected through cognitive tests, may benefit from being included in our research due to self-reported complaints via a formal interview and/or feedback from those around them (e.g., family, friends). This would overcome the problem researchers face in validating acquired patients’ self-identified prosopagnosia status given how rare the condition is and how few self-identified cases (i.e., 14%) fail to display abnormality on cognitive tests. We recently validated developmental cases’ self-reported status by pooling the excluded cases’ data to show they exhibit objective group-level impairments on multiple cognitive tests. Unfortunately, this is not possible here due to the difficulty in finding acquired cases who failed to meet objective criteria in sufficient numbers.

To further help address this issue, it would be timely for acquired prosopagnosia researchers to include such potential or borderline individuals in their papers going forward so we can pool these cases’ data to ascertain whether they exhibit objective impairments at the group level. We appreciate that the inclusion or exclusion of such potential AP cases may depend on the aims of the research study at hand. However, we recommend their inclusion, even if it is in solely in the interests of transparency (i.e., how many potential APs were initially tested, and how many failed to meet objective criteria) so that we may better understand this almost entirely unstudied group. After enough cases are reported on, we will be able to identify group-level impairments to validate their self-reported status, as we have done in prior DP work [27,34].

We recognise that some authors believe self-report alone should never be used as a classification tool [7,30,65]. For those who wish to follow some form of the DSM-5 approach to neurocognitive disorders, we would recommend the Liberal DSM-5 approach over the Adapted DSM-5 method. The Liberal DSM-5 included more AP cases (i.e., 86–100%) than the Adapted DSM-5 (71–82%). However, it is important to note that even the Liberal DSM-5 approach introduced redundancy, as using the FFT alone at the −2 SD level produced the same rates of exclusion from research. Based on our findings, we suggest that where researchers want to recruit AP cases, verbal self-report may be helpful to initially identify the extent of the full AP distribution, i.e., complaints that the patient has difficulties recognising people they should know. This range of initially identified cases should be reported on by researchers with some measures of their objective abilities (e.g., perception, and familiar and unfamiliar face memory). From this, researchers could include cases in their research using structured self-report via a standardised questionnaire, such as an adapted form of the prosopagnosia index symptom measurement and a famous faces test (i.e., at the −2 SD level), to classify cases as objectively impaired. These simple criteria will allow more AP cases to be included in research studies while also allowing us to learn more about cases who fail to meet the criteria.

### 4.2. Conceptualising Prosopagnosia

How should we conceptualise prosopagnosia? Are the developmental and acquired forms qualitatively similar? One way of answering these questions is to compare the patterns of abnormalities detected on cognitive tests. While we urge caution when interpreting such comparisons, due to our small and exploratory self-identified AP sample, we do believe this will be of interest. Individual AP scores identified as atypical at the −2 SD level in our cases on the CFPT at 18%, the CFMT at 55%, and FFT at 82%. These rates are remarkably close to those found in self-identified DP cases (CFPT: 23%, CFMT: 44%, FFT: 75%, [27]). Moreover, the self-identified DP and AP cases exhibited qualitatively similar patterns of impairments, with the mean z-scores revealing difficulties in perception (i.e., CFPT: AP = −0.77, DP = −1.28; [27]) that are smaller than those found in unfamiliar memory (i.e., CFMT: AP = 2.05, DP = −1.79; [27]), which, in turn, are smaller than those in familiar face memory (i.e., FFT: AP = −4.7, DP = −3.1; [27,32]). This pattern of results suggests that both acquired and developmental prosopagnosia are conditions in which face memory deficits are most apparent, in contrast to troubles with face perception. Moreover, face memory difficulties seem most pronounced as faces become more familiar. This gradient might reflect the underlying observation that neurotypical human face recognition performance is remarkably good for familiar faces but surprisingly poor for unfamiliar faces [34,66], suggesting that the expert system for familiar face recognition is more easily detected as compromised in self-reported prosopagnosia cases. This pattern of results might also be related to familiar face recognition being the most obvious and striking sign of face recognition impairment to the individual (especially when one had this ability and then lost it), while it may be much more difficult to gain self-awareness of unfamiliar face-processing ability.

### 4.3. The Use of the CFMT as an Inclusion/Exclusion Tool in Prosopagnosia Research

It is possible that our failure to detect any objective CFMT impairment here in some cases could have been due to the fact these individuals do not have prosopagnosia. However, some of these cases were highly impaired on the FFT, demonstrating objective troubles that are likely to affect them in daily life. Thus, these cases show that the CFMT is potentially problematic because most researchers are using it to include or exclude acquired and developmental prosopagnosia [30,32,34,35,36]. Moreover, it was the sole cause of exclusion here in every AP individual when we required atypicality on the FFT and CFMT. Similarly, the CFMT has lower power for detecting abnormality than a familiarity adjusted FFT, e.g., [32]. Given that the CFMT fails to classify around 50% of self-reported acquired and developmental cases, we must ask if it is likely that these people have a failure of meta-cognition as some have suggested [67]. Or does the CFMT lack the sensitivity for including all objectively impaired cases in our work?

We suggest that the CFMT as a classification tool for prosopagnosia skews the profile of those who will be accepted as having prosopagnosia towards those with difficulties with unfamiliar face processing and face perception. By contrast, when we look at individuals who self-report face-processing difficulties (APs and DPs), they tend to exhibit their most severe problems in familiar face memory [27,34]. If people with prosopagnosia are primarily suffering problems recognising individuals personally known to them, such as co-workers, neighbours, friends, and family members, then it is unsurprising that the CFMT fails to identify abnormality in many cases, as their primary complaint is familiar face recognition. By contrast, the FFT indexes the cognitive ability that is clearly most impaired in prosopagnosia, namely, familiar face recognition. This means that we must be cautious about using the CFMT as an inclusion/exclusion criterion in acquired prosopagnosia but rather as a complimentary tool to assess which aspects of face processing are impaired. It is important to note that the CFMT’s classification of acquired prosopagnosia may yet be improved by the inclusion of response times, as has been achieved in DP [68]; unfortunately, we could not test this here, as our software program did not record the participants’ response times. We anticipate, given that AP cases can exhibit elevated response times when identifying faces (e.g., [69]), that it seems likely that incorporating this data may cause the CFMT to detect higher rates of abnormality in AP.

### 4.4. Limitations

This study has limitations that we must mention. We did not systematically have access to detailed lesion information, time since injury, visual-field assessment, broader object-recognition measures or comprehensive neuropsychological profiles. The participants reported a marked acquired deterioration in face recognition following brain injury or stroke, and some discussed neurological assessment and brain imaging in informal correspondence with the research team, but these details were not collected systematically enough to provide independently verified clinical characterisation. Consequently, the present data cannot establish whether the participants’ reported face-recognition difficulties were entirely face-specific or whether broader visual or cognitive impairments also contributed in some cases.

This limits our conclusions concerning the neurological cause of the participants’ difficulties, but it does not preclude our observations about the discrepancy between cognitive testing and self-report examined here. We asked how individuals reporting a marked acquired deterioration in everyday face recognition performed on tests commonly used to include or exclude acquired prosopagnosia from research. Even if broader impairments contributed to some participants’ everyday difficulties, this would not straightforwardly explain why they sometimes performed within conventional ranges on laboratory face-processing measures—in fact, mid-level visual difficulties are usually accompanied by *poorer* face-processing performance [70]. We therefore reject suggestions that these difficulties should be an *exclusion* criterion, as such individuals still experience the defining problems associated with prosopagnosia, i.e., difficulties recognizing faces in daily life. If anything, the correlation between broader mid-level visual functioning and face recognition troubles in DP are potentially reflective of the fact that both utilize shared cognitive resources (Burns et al., 2019 [71]). Future work should therefore examine the discrepancy between cognitive tests and self-report in larger samples with detailed neurological, visual and neuropsychological characterisation.

The use of FFT also requires consideration of cultural and demographic familiarity. This issue was reduced in the present study because all the participants were White British and were over 35 years of age and therefore completed an older-adult version of the FFT, which contained public figures selected to be familiar to that age group. In addition, the scores were adjusted according to whether each participant reported knowing the individual depicted. These features make unfamiliarity with the stimulus identities unlikely to provide a simple explanation for the present pattern of results. Nevertheless, the diagnostic sensitivity observed here should not automatically be assumed to generalise to other countries or cultural groups. Famous-face measures used internationally would require culturally and age-appropriate identities and corresponding normative data rather than direct application of the British version used here.

Although the FFT seems to be the most sensitive tool for detecting impairment in people who self-identify as having acquired prosopagnosia, it is important to note 2/14 cases (14%) failed to exhibit impairment. It is unclear whether this is due to the FFT lacking the sensitivity to detect impairment in AP or whether these cases have difficulties realizing their face recognition skills are intact. As noted, Lowes et al. have employed response times to improve the CFMT’s sensitivity for detecting impairment in DP [68]. We expect that incorporating such data into the accuracy rates will lead to similar improvements in detection rates in AP.

## 5. Conclusions

Experimental test-based approaches to identifying cognitive impairments fail to detect abnormality in many self-identified developmental prosopagnosia cases [27,30,32,33,34]. We have shown the same issues in self-identified acquired prosopagnosia apparent here, too, where many individuals fail to meet criteria on two tests when using the Adapted DSM-5 and traditional approaches to classification. These problems were partially, but not entirely, resolved using the Liberal DSM-5 approach. However, the FFT alone was as effective in classifying acquired prosopagnosia as the Liberal DSM-5 method, and so, if an objective assessment is deemed necessary, then it should be the only test used for inclusion criteria. If a patient fails to meet the criteria using this task, then atypicality on an adapted PI20, coupled with reports of highly abnormal face recognition failures (e.g., co-workers, friends, family members) should be employed. Only by listening to these individuals’ complaints of their face recognition failures can we hope to learn more about these cases’ problems and why cognitive tests fail to detect atypicality [32].

## Figures and Tables

**Table 1:** Distinct approaches to identifying prosopagnosia for research.

Inclusion Criteria	Tasks/Measures Required for Assessment	Cutoff for Inclusion	Notes
**Traditional:** Two Tests	FFT and CFMT	−2 SDs	N/A
**Traditional:** Two out of three	Two identity tests	−2 SDs	No restrictions on tests used, but requires impairment in two of three (e.g., CFMT, FFT and CFPT)
**Adapted DSM-5**	Two identity tests	−1 SDs	Must be two memory tests (DeGutis et al., 2023 [30])
**Liberal DSM-5**	Two or one identity tests	Two tests −1 SDs or Single Test −2 SDs	No restrictions

We provide details on two traditional approaches (i.e., impaired on the CFMT and FFT, or impaired on two out of CFPT, CFMT and FFT) taken by many researchers in the past: the Adapted DSM-5 approach (DeGutis et al., 2023 [30]), and the Liberal DSM-5 approach that adds the inclusion option of impaired at the −2 SD level on a single test [34]. We have bolded the column headers and criteria names for clarity.

**Table 2:** Neuropsychological test results of the 14 self-identified AP cases.

CFMT & FFT −2 SD Inclusion for Research?	Two of Three −2 SD Inclusion for Research?	Adapted DSM-5: −1 SD on FFT AND CFMT Inclusion for Research?	Liberal DSM-5: −1 SD on Two Tests OR −2 on One Test Inclusion for Research?	Age	Sex	CFMT	CFPT Up	FFT	PI20
No	No	No	Yes	65	Female	−0.24	0.48	**−2.38**	**−3.32**
No	No	No	Yes	48	Female	−0.37	−0.72	**−4.45**	**−3.04**
No	No	Yes	Yes	62	Female	−1.13	0.15	**−2.61**	**−2.86**
No	No	Yes	Yes	53	Female	−1.13	−1.38	**−7.59**	**−2.67**
No	No	Yes	Yes	73	Female	−1.25	0.04	**−4.70**	**−4.7**
Yes	Yes	Yes	Yes	47	Male	**−2.27**	1.02	**−3.83**	**−3.69**
Yes	Yes	Yes	Yes	39	Male	**−2.39**	−1.71	**−5.44**	**−3.69**
Yes	Yes	Yes	Yes	49	Male	**−2.65**	−1.38	**−8.51**	**−3.59**
Yes	Yes	Yes	Yes	70	Male	**−2.77**	−0.83	**−4.72**	**−2.67**
Yes	Yes	Yes	Yes	68	Male	**−4.04**	**−2.14**	**−7.80**	**−2.03**
Yes	Yes	Yes	Yes	56	Female	**−4.29**	**−2.03**	**−4.29**	**−3.13**
No	Unclear	No	Unclear	45	Male	X	X	−0.09	**−2.58**
No	Unclear	No	Unclear	56	Male	X	X	−0.03	**−2.12**
Unclear	Unclear	Unclear	Yes	76	Male	X	X	**−9.41**	**−4.6**

Columns on the right indicate Famous Faces Test (FFT), Cambridge Face Memory Test (CFMT), Cambridge Face Perception Test upright scores (CFPT Up) and the prosopagnosia index (PI20). Bold indicates impairment of −2 SDs using norms in the literature (CFMT: [31]; CFPT: Duchaine et al., 2007 [33]) or from our control sample (FFT). The first four columns indicate inclusion criteria status using the traditional two-tests approach (−2 SD on CFMT and FFT), the two-out-of-three tests approach (−2 SD in two of CFPT, CFMT and FFT), the Adapted DSM-5 (−1 SD on FFT and CFMT) and a more liberal approach to the DSM-5, which was recently suggested by Burns (2024) [17] (−1 SD on two tests, or −2 on a single test). We use colour as a visual aid to make it easier to identify cases that met criteria (green), did not (red), or where it was ambiguous (orange).

## Data Availability

Data can be found on the OSF at the following link: https://osf.io/4q7az/files/kj5xf (accessed on 27 August 2026).
